# The use of image analysis to study the effect of moisture content on the physical properties of grains

**DOI:** 10.1038/s41598-024-60852-7

**Published:** 2024-05-22

**Authors:** Łukasz Gierz, Mustafa Ahmed Jalal Al-Sammarraie, Osman Özbek, Piotr Markowski

**Affiliations:** 1 Institute of Machine Design, Faculty of Mechanical Engineering, Poznan University of Technology, ul. Piotrowo 3, 60-965 Poznan, Poland; 2 Department of Agricultural Machinery and Equipment, College of Agricultural Engineering Sciences, University of Baghdad, Baghdad, Iraq; 3 Department of Agricultural Machineries and Technologies Engineering, Faculty of Agriculture, Selçuk University, 42250 Konya, Turkey; 4 Department of Heavy-Duty Machines and Research Methodology, University of Warmia and Mazury, ul. Oczapowskiego 11, 10-719 Olsztyn, Poland

**Keywords:** Physical properties, Image analysis, Moisture content, Overall dimensions, Artificial neural network, Mechanical engineering, Information theory and computation

## Abstract

Designing machines and equipment for post-harvest operations of agricultural products requires information about their physical properties. The aim of the work was to evaluate the possibility of introducing a new approach to predict the moisture content in bean and corn seeds based on measuring their dimensions using image analysis using artificial neural networks (ANN). Experimental tests were carried out at three levels of wet basis moisture content of seeds: 9, 13 and 17%. The analysis of the results showed a direct relationship between the wet basis moisture content and the main dimensions of the seeds. Based on the statistical analysis of the seed material, it was shown that the characteristics examined have a normal or close to normal distribution, and the seed material used in the investigation is representative. Furthermore, the use of artificial neural networks to predict the wet basis moisture content of seeds based on changes in their dimensions has an efficiency of 82%. The results obtained from the method used in this work are very promising for predicting the moisture content.

## Introduction

Corn (*Zea*) is classified as one of the three most important cereal crops in the world. For humanity, of the 6 species^1^ of corn, the most important in economic terms is common corn (*Zea mays L.),* also called the type species^2^. The grains (seeds) of individual corn varieties differ in size, color, shape, and texture. The most common varieties are yellow, red, and white corn kernels, which constitute an important traditional food, animal feed, and industrial raw material that play an important role in agricultural production^3,4^. In turn, beans belong to the legume family. In the world, it is produced and consumed mainly in the form of dry beans and green beans. Legumes are the main source of proteins and nutrients such as oil, fiber, starch, vitamins, and minerals^5,6^^.^ Corn is grown in more than 170 regions of the world, with the main growing regions being North America, Asia, and South America. Corn production in the United States (USA), China, Brazil and Argentina accounts for up to 64.63% of world production^7^. Meanwhile, world bean production reached Asia, Africa and the Americas (North, Central, and South) with 49.72%, 24.40%, and 24.36% respectively^8^. Sustainable food systems are required to ensure sustainable food production that can meet the growing demand for a world population expected to reach 9.8 billion people by 2050^9^. In today's competitive market, manufacturers must offer products classified by physical characteristics such as appearance, size, color, and internal health status. Moreover, identifying the varieties of grains helps farmers in selecting the right grains to sow and marketing because the grains they sell thus sell will be of the basic standard of marketers^10^. The physical properties of grains are important for equipment design and analysis of product behavior during agricultural processes such as planting, harvesting, threshing, cleaning, classification, and processing. The main grain dimensions are useful for selecting screen spacers and calculating the grinding force (reduction) when reducing size. They can also be used to calculate the surface area and size of grains, which is important when modeling grain drying, ventilation, heating and cooling processes^11^. The manual process of grain sorting is time-consuming and inefficient, especially in the case of large production volumes. For these reasons, new methods have been introduced, such as vision systems for classification, sorting, and measurement. Due to increasing consumer demand and technological progress, industries are engaged in the automation of processing steps such as grading, sorting, packaging, determining the quality of fruits and vegetables, automation and design of handling mechanisms, processing, peeling, etc. Physical properties such as weight, volume, surface area, bulk density, etc. must be known, therefore, it is necessary to check the relationship between them^12^. Alamery and Al-Badri^13^ developed mathematical models to predict grain mass and size based on grain length, width, and thickness. Image-processing techniques are used after agricultural produce is harvested to ensure food quality, hygiene, and processing suitable for human consumption.

The selected physical characteristics of seeds are an important parameter in the assessment of food quality. Consumers expect agricultural products of the same size and characteristic shape. Eboibi and Uguru^14^ used statistical analysis such as analysis of variance (ANOVA) and Duncan's multiple range test to investigate the physical properties of bean varieties. Yenge et al.^15^ determined the physical and mechanical properties of corn kernels as a function of moisture content using standard techniques. Kruszelnicka^16^ examined the physical and mechanical properties of corn grains from the point of view of designing mechanical processing systems. Today, the latest research on cereal products is carried out using machine vision systems and image processing technologies. Using the methods and equipment mentioned, products are examined for many physical properties, such as color^17^, texture, quality^18^, and size. Image processing uses various mathematical and numerical models to associate one or more properties and also to predict various physical properties of products. Several studies have been conducted on the mathematical and numerical relationship between the physical properties of fruits. Ghabel et al.^19^ describe the relationship between mass and geometric mean diameter. The surface area and volume of models with different shapes can be measured by estimating three mutually orthogonal axes^20^. Grain classification has been studied by several researchers using image analysis. Shahin and Symons^21^ used color and grain size using a planar imaging system to determine color classification. In the case of Sicilian and Canadian lentil varieties, color characteristics were found to be good predictors for variety recognition. Kılıç et al.^22^ developed a bean classification system based on seed size and color. The results showed that this method allowed for the correct classification of seeds. Guevara-Hernandez and Gil^23^ developed an automated vision system for classifying wheat and barley grains. The results of verification of the developed system showed that accuracy exceeding 99% can be achieved when extracting morphological, color, and textural features from grains. Tahir et al.^24^ successfully used machine learning algorithms to extract features from images of western Canadian durum wheat, western Canadian red spring wheat, and barley to investigate the effect of moisture content on the ability to classify the physical properties of the imaged grains. Wilson et al.^25^ measured the grain size distribution of wheat starch using image analysis and laser diffraction techniques. Arasan et al.^26^ used image analysis methods to determine the grain size distribution (granulometric composition). The literature also indicates that the physical properties of cereal grains depend mainly on the water content^27^. Research on predicting the impact of moisture content based on the physical properties of grains (seeds) using image processing technology has not been precisely defined; therefore, there is a gap in the state of knowledge in this area, which was decided to fill. The article sets out three specific goals. The first was to determine the effect of humidity on the physical properties of beans and corn (geometric dimensions), such as length, width, and thickness. The second was to assess the usefulness of the developed method using the image analysis technique to determine the geometric dimensions of the grain. The third goal was to use an artificial neural network (ANN) to predict the moisture content of beans and corn grains based on their geometric dimensions.

## Materials and methods

### Sample preparation

It should be noted that for the purposes of the investigation, the authors used varieties of crop plants sown in Poland. It is also worth noting that, based on statistical data from 2021, Poland became the third corn producer in the European Union, after France and Romania, with a 11% share in EU production (https://www.fao.org/faostat/en). The research material consisted of beans of the Eureka variety (*Phaseolus coccineos L.*) and corn seeds of the Gramatura variety (*Zea Mays L.)* All research material came from PlantiCo Zielonki Sp. z o. o. Spójna, Breeding and Seed Plant in Nochowo, located in Greater Poland Province. Based on the announcement of the Marshal of the Sejm of the Republic of Poland about the Legal Protection of Plant Varieties of January 22, 2021 (Journal of Laws of 2021, item 213) and the breeder's declaration that the indicated varieties: Eureka (beans), Grammaturia (corn) are protected by law by the breeder; the authors have received this permission. The breeder agreed to provide the above-mentioned plant material, and it's use is in complied with the national guidelines of the Main Seed Warehoue, a production plant in Nochowo. With the consent of the breeder, the authors could use the obtained plant material only for scientific research purposes, including research on, among others, oversize dimensions.

The samples were manually cleaned to remove foreign bodies, dust, dirt, broken and immature grains. The weight of one sample was 500 g, which was determined using an electronic scale RADWAG PS 4500/X with an accuracy of 0.01 g. The initial moisture of the samples, which was 9%, was determined using a digital grain moisture meter AR991 with a measurement range of 7.5–50% and measurement accuracy of 0.1%, made in China. The grains were subjected to another measurement procedure after moistening to the level of 13% and 17% by adding measured amounts of filtered water *M*_*w*_ according to (Eq. 1), also used in other works^28,29^.

1 $${M}_{w}=\frac{({W}_{2}-{W}_{1})}{100-{W}_{2}}.{M}_{p}$$

where: ${M}_{w}$—the amount of water needed to increase the moisture content (g), ${M}_{p}$—the weight of the sample (g), *W*_1_—the initial moisture content of the sample (%), *W*_2_—the assumed moisture content of the sample (%).

After adding a certain amount of water, the seeds were closed in polyester string bags and stored at (5 °C) in the refrigerator for 7 days to obtain an even distribution of moisture in the seeds. The humidity stabilization time was determined on the basis of studies conducted previously^30–33^, in which it was 5 or 15 days. Then the required amounts of seeds were taken from string bags to perform measurements, and the remaining seeds were used to control the wet basis moisture content.

The amount of moisture in a biological material can be expressed in terms of wet weight (wet weight) or dry weight (dry weight), expressed as a decimal or percentage^34^. For the purposes of this research, the wet basis moisture content expressed as a percentage of *M*_*wb*_ was determined based on Eq. (2) ^34^, defined as the mass of moisture contained in the seeds per unit mass of undried seeds:

2 $${M}_{wb}=\frac{{W}_{o}-{W}_{d}}{{W}_{o}}\cdot 100$$

where: *M*_*wb*_—wet basis moisture content expressed as a percentage (%), *W*_*o*_—initial weight of undried seeds (g), *W*_*d*_—mass of dry seed (g).

### System methodology

The research methodology consists of six steps: image capture of each grain, image processing, grain edge detection (find contours from image of each grain), data acquisition and description, data preservation, and prediction of moisture content on a wet basis.

The three main dimensions, length (*L*), width (*W*) and thickness (*T*), were measured for 100 randomly selected kidney beans and maize (A group of grains was taken from one sample and then it was divided again until we reached 100 grains) using the proposed system.

### Preparation of materials for camera tests

**Figure 1:**
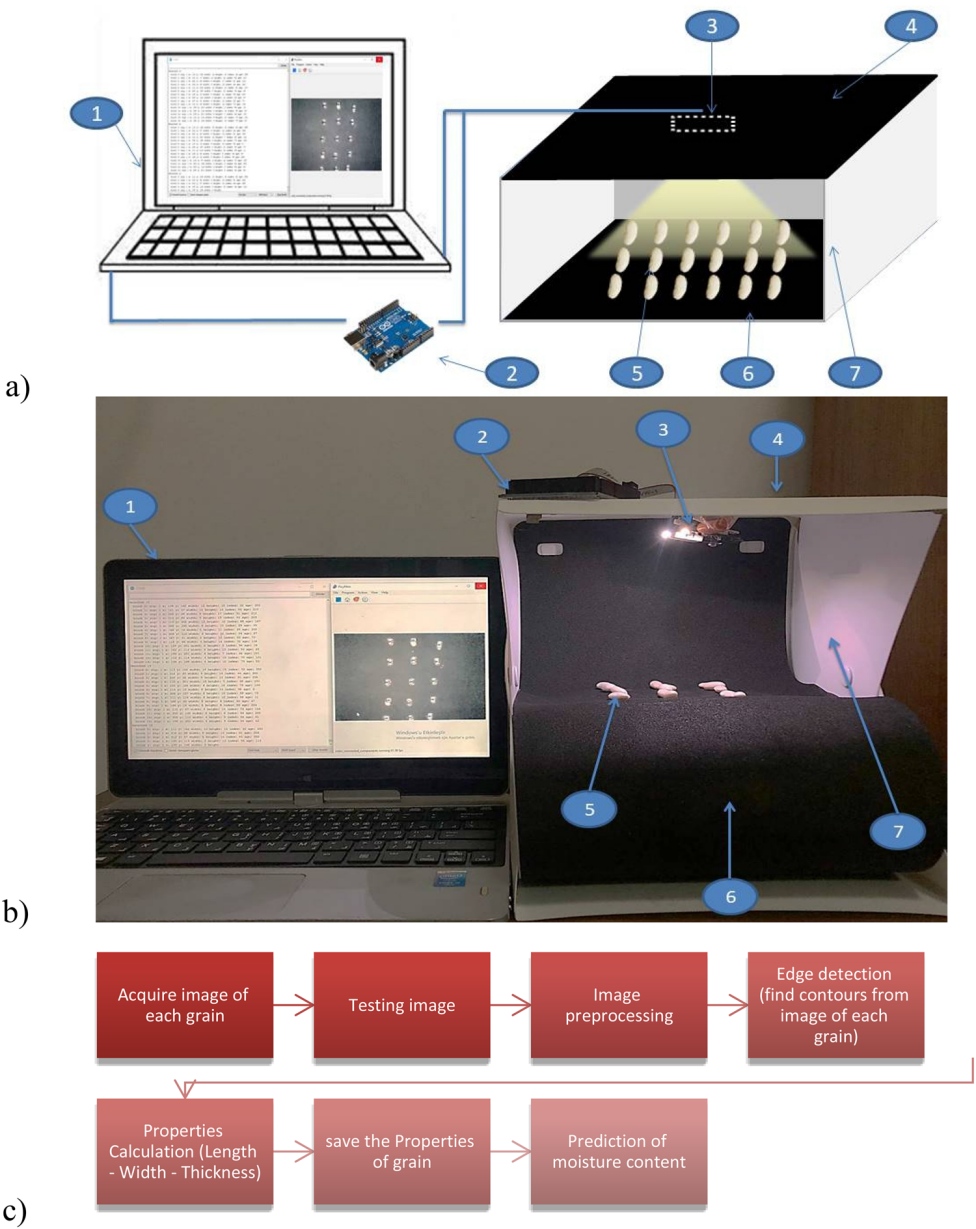
Measurement system, (**a**) schematic diagram: (1) laptop, (2) control unit (Arduino UNO), (3) pixy2 camera, (4) cover, (5) grains, (6) black plate, (7) grain chamber: (**b**) real view; (**c**) block diagram.

Using the pixy2 camera, the main grain dimensions, such as length (*L*), width (*W*), and thickness (*T*), were measured for 100 randomly selected beans (seeds) of beans and corn. The pixy2 camera is characterized by low purchase cost and ease of use. The camera used contains an Omni Vision OV9715 sensor, 1280 × 800, 1/4. It can process 50 images per second (20 ms per image). It can be connected to a computer via the USB port. Additionally, it is characterized by a very low weight of 27 g and a low current consumption of 140 mA^35^. The camera is connected to the Arduino Uno controller using the serial communication protocol, thanks to which the camera takes photos from different angles. The camera was successfully used in the analysis of fruit images^36^. An LED camera light source was used, the camera light intensity of which is 341 Lux. The light intensity of the camera was measured using a digital lux meter. Measuring range 0.1 ~ 200,000 lx, 0.01 ~ 20,000 Fc, resolution: 0.1 lx/0.01 Fc, accuracy ± 4%. The camera was mounted vertically above the measuring table (grain). This mounting provided a field of view of 60 degrees horizontally, 40 degrees vertically from a height of 15 cm. Figure 1 shows the measurement system.

To obtain accurate measurements, an attempt was made to reduce the shadow from the program settings. The pixy2 camera was calibrated by entering images of different grains. After preparing the program with different grain images, the test environment was adjusted by monitoring the lighting intensity. The lighting intensity should be bright enough to allow photos to be taken with the required sharpness. The image capture unit was isolated from room light and sunlight, which had a positive effect on the calibration process^37^. In each measurement cycle, the grains were arranged in three columns and six rows. During the measurement, a space was deliberately left between the grains because the grain arrangement greatly influences the overlap of the image frames, which affects the measurement accuracy. It has been proven that placing samples too close to each other, in our case grains (seeds), causes cross-contamination during measurement^38^.

### Grain dimensions measurement system

**Figure 2:**
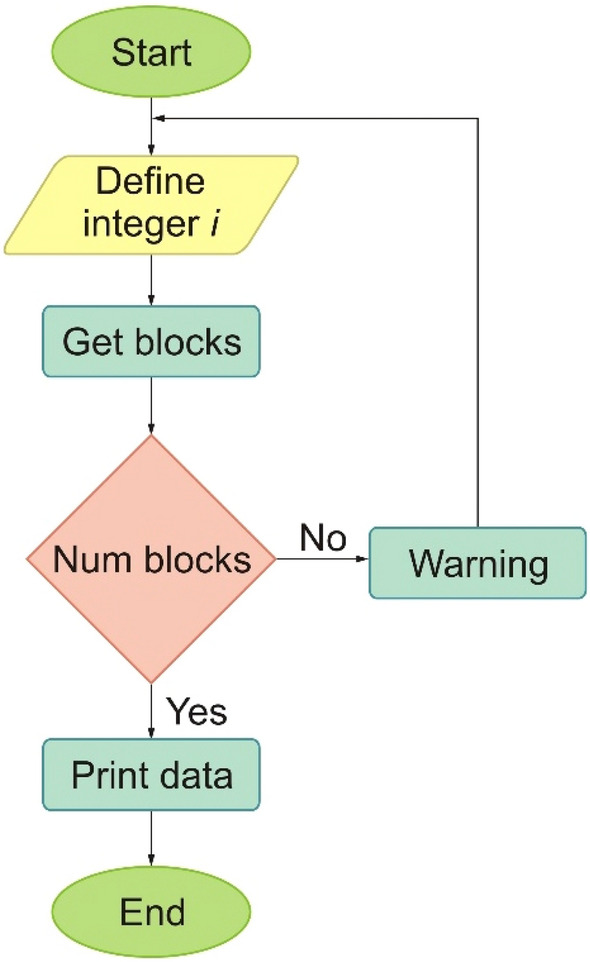
System algorithm.

A computer vision system consists of two units: (1) an image processing unit and (2) a pattern recognition unit. The system is controlled by a microcontroller. The system captures an image of the grain (seed) and sends it to the image analysis processor, and the physical properties of the various grains are calculated. Figure 2 shows the system algorithm. The system code was written in C++.

To process the images recorded with the pixy2 camera, the PixyMon program was used. The program is easy to use and sends the necessary data to the control unit. To measure the main dimensions, the edge of the grain must be detected by detecting the edge pixels in the image. Edge pixels are a group of points (pixels) on a curve that separate adjacent points (pixels) or points (pixels) on the other side of the curve that differ in brightness^39^. The beans were placed in front of the Pixy2 camera and the ‘Set Caption’ option was selected. This option allows you to select the desired grain displayed on the screen in a rectangle of the desired size. PixyMon then highlights the entire grain. PixyMon divides each seed into a “block” that assigns a signature. This block signature can be sent to the control unit (Arduino UNO), e.g. the height, width, and distance of the block. You need a computer to distinguish the blocks. These data are stored in the memory card of the Pixy2 camera after it is disconnected from the computer.

After the analysis, we receive the following data: Block (this is the position of the block in the table), sig (signature number 1-7), x and y (coordinates of the block in the visible frame), width, height (length), index (unique number assigned to the object on the display) and age (the number of frames in which a given block was viewed, 1-255).

The measurement dimensions of the pixy2 camera are similar to the human eye in that distant objects are perceived as having small dimensions and increase as the distance between the camera and the object shortens. Therefore, when accurately calculating the main dimensions, the distance of the camera from the grains is calibrated and the results obtained from the camera are compared with those obtained by manual calculation using a caliper. A caliper with an accuracy of 0.02 mm was used for manual measurement. After adjusting the distance of the camera lens from the measuring table, the identified dimensions are divided by 10 to obtain greater measurement accuracy.

### The structure of an artificial neural network

**Figure 3:**
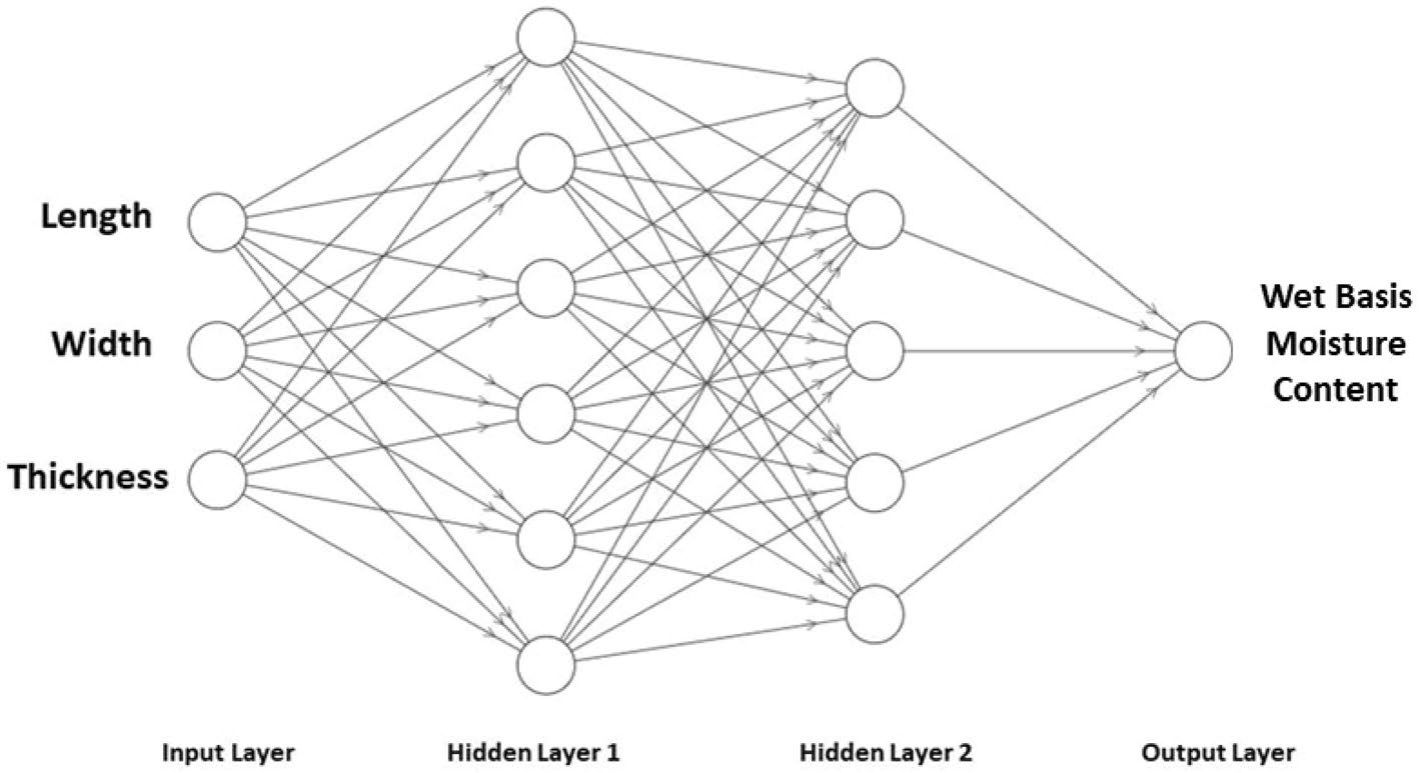
Neural network test.

When creating artificial neural networks of the multilayer perceptron (MLP) type, the first step is to design training sets. To obtain appropriate results, it is very important to determine the characteristics being studied and the number of training instances. From the proposed system, grain size data were obtained, indicating the wet basis moisture content. An artificial neural network was designed, which includes an input, hidden, and output layer. The number of hidden layers, including the number of neurons and the type of activation function in the hidden and output layers, was determined using the Weka tool. The Weka tool includes a set of visualization tools and algorithms for data analysis and predictive modeling. This tool is publicly available and has easy access to user interfaces. The program is entirely implemented in the Java programming language, so it works on almost any modern computer platform. The neural network contained an input layer that contained the main dimensions of the grains (length, width, and thickness), two hidden layers for each variable with a different number of neurons (6.5), while the output layer contained the wet basis moisture content (see Fig. 3 for a diagram). The quality of the obtained networks was assessed based on the error of the training, testing, and verification sets, and then the root mean square error was determined. During the training process of each neural network, the data was randomly divided into three groups: training sets,, test sets and validation sets. The training data set used in the learning process consisted of 80%, test sets 10%, and validation sets 10%. To define the performance measure, the definition defined in the previous work^40^ was used.

**TP rate:** Which represent positive samples. The TP rate equation is:

3 $${{{\text{TP}}} \mathord{\left/ {\vphantom {{{\text{TP}}} {\left( {{\text{TP1}}\, + \,{\text{FN1}}} \right)}}} \right. \kern-0pt} {\left( {{\text{TP1}}\, + \,{\text{FN1}}} \right)}}$$

**FP rate:** A positive error, which is considered a positive sample, but is actually a negative sample. The FP rate equation is:

4 $${{\text{FN1 }} \mathord{\left/ {\vphantom {{\text{FN1 }} {\left( {{\text{TP1}}\, + \,{\text{FN1}}} \right)}}} \right. \kern-0pt} {\left( {{\text{TP1}}\, + \,{\text{FN1}}} \right)}}$$

**Precision**: The rate of correct cases for a given category, and correctly predicted by the classifier. The precision equation is:

5 $${{{\text{TP1}}} \mathord{\left/ {\vphantom {{{\text{TP1}}} {\left( {{\text{TP1}}\, + \,{\text{FP1}}} \right)}}} \right. \kern-0pt} {\left( {{\text{TP1}}\, + \,{\text{FP1}}} \right)}}$$

**Recall:** The ratio of the truly integer number in the retrieval results in the integer number in the entire data set (recovered and unrecovered). The recall equation is:

6 $${{{\text{TP1}}} \mathord{\left/ {\vphantom {{{\text{TP1}}} {\left( {{\text{TP1}}\, + \,{\text{FN1}}} \right)}}} \right. \kern-0pt} {\left( {{\text{TP1}}\, + \,{\text{FN1}}} \right)}}$$

**F-measure:** is the discrepancy between the precision P and R of the weighted mean harmonic index. The F-Measure equation is:

7 $$\frac{{\text{TP}}1\cdot {\text{TN}}1-{\text{FP}}1\cdot {\text{FN}}1}{\sqrt{({\text{TP}}1+{\text{FP}}1)({\text{TP}}1+{\text{FN}}1)({\text{TN}}1+{\text{FP}}1)({\text{TN}}1+{\text{FN}}1)}}$$

**ROC area:** The area under the ROC curve (AUC) provides another way to evaluate the average performance of a model.

PRC Area: Use exact registration formulas to complement ROC equations to obtain the full spectrum during analysis and selection.

**Accuracy:** Correctly classified cases. The accuracy equation is:

8 $$\frac{{{\text{TP}}1 + {\text{TN}}1}}{{{\text{TP}}1 + {\text{TN}}1 + {\text{FP}}1 + {\text{FN}}1}}$$

where: TP1– positive true, FP1– positive false, TN1– negative true, FN1– negative false.

### Methods of developing research findings

The research results were statistically processed using the following procedures:

1. Determining basic measures of position and dispersion of measurement results of individual physical properties of grain (length, width, and thickness) obtained using image analysis techniques and calipers;
2. Verification of hypotheses assuming that the empirical distributions of the values of individual geometric characteristics of beans and corn grains (measured with individual measuring instruments and dependent on humidity) are consistent with the normal distribution. Calculations were carried out using the following tests: W Shapiro–Wilk and Komogorow-Smirnow (with Lilliefors' correction) and χ2-Pearson;
3. Assessment of the homogeneity of variance of the measured feature (Levene’s test) if its distribution is consistent with the normal distribution;
4. Comparison of the the significance of differences between the average values of the individual physical properties of beans and corn grain:
  - Measured with different t-Student measuring devices, when the measured features had a normal distribution and with the Mann–Whitney U test, when the distribution of features was not compliant with the normal distribution,—the assumption of the Student's t-test was not satisfied;
  - Depending on humidity; If the distribution of the measured characteristic was consistent with the normal distribution and there was homogeneity of variance, one-way analysis of variance (ANOVA) was used for the calculations. Otherwise, the nonparametric Kruskal–Wallis test was used. Furthermore, when statistically significant differences were demonstrated between the mean values ​​of the measured trait, ‘post-hoc’ tests were performed. In the first situation, when it was concerned with ANOVA analysis, the significance of differences was checked with tests of different sensitivity, that is, Fisher, Scheffe, HSD Tukey and Duncan, and if it was related to the results of the Kruskal–Wallis test, the option of multiple comparisons was used for mean "rank" values. The purpose of these analyzes was to distinguish so-called homogeneous groups.

The calculations were performed at the significance level of α = 0.05, using the STATISTICA PL statistical program.

## Results and discussion

### Dimensions of the main grains

Two methods were used in the investigation to measure the main dimensions of corn and bean grains: the first using the Pixy2 camera and the second using a caliper (control method). First, the distribution of individual grain dimensions (length, width, and thickness) measured with various devices was checked to be consistent with the normal distribution. Table 1 presents measures of the significance distribution of individual geometric features (length, width, and thickness) of the grain, which were measured with various measurement tools.

**Table 1:** Parameters of significance distribution of individual geometric features (length, width, and thickness) of grain measured using various measuring instruments.

Geometric dimension	Measurements made with the new method					Control measurements				
	N	maks D	Lillief. *p*	S-W *W*	*p*	N	maks D	Lillief. *p*	S-W *W*	*p*
Bean grain–moisture 9%										
Length	20	0.17.215	*p* < 0.15	0.93.983	0.23.797	20	0.15.571	*p* > *0.20*	0.93.168	0.16.636
Width	20	0.19.195	*p* < 0.05	0.89.182	0.02.904	20	0.19.111	*p* < *0.05*	0.89.360	0.03.134
Thickness	20	0.13.003	*p* > 0.20	0.95.132	0.38.754	20	0.13.720	*p* > *0.20*	0.94.666	0.31.923
Bean grain–moisture 13%										
Length	20	0.15.786	*p* > *0.20*	0.91.764	0.08.925	20	018.059	*p* < *0.10*	0.94.929	0.35.638
Width	20	0.24.761	*p* < *0.01*	0.81.674	0.00.155	20	0.28.897	*p* < *0.01*	0.71.324	0.00.006
Thickness	20	0.16.805	*p* < *0.15*	0.93.003	0.15.464	20	0.16.130	*p* < *0.20*	0.90.130	0.04.361
Bean grain–moisture 17%										
Length	20	0.16.947	*p* < *0.15*	0.92.596	0.12.910	20	0.18.269	*p* < *0.10*	0.94.184	0.25.970
Width	20	0.29.726	*p* < *0.01*	0.89.583	0.03.447	20	0.16.764	*p* < *0.15*	0.92.676	0.13.372
Thickness	20	0.20.909	*p* < *0.05*	0.88.719	0.02.388	20	0.20.312	*p* < *0.05*	0.89.624	0.03.508
Maize grain–moisture 9%										
Length	20	0.11.296	*p* > *0.20*	0.95.375	0.42.769	20	0.18.502	*p* < *0.10*	0.93.522	0.19.446
Width	20	0.12.551	*p* > *0.20*	0.96.673	0.68.487	20	0.25.266	*p* < *0.01*	0.92.313	0.11.386
Thickness	20	0.22.525	*p* < *0.05*	0.90.765	0.05.749	20	0.22.988	*p* < *0.05*	0.92.565	0.12.732
Maize grain–moisture 13%										
Length	20	0.14.150	*p* < *0.10*	0.95.595	0.13.045	20	0.15.089	*p* < *0.05*	0.95.729	0.14.469
Width	20	0.18.366	*p* < *0.01*	0.94.163	0.04.308	20	0.22.554	*p* < *0.01*	0.92.432	0.01.186
Thickness	20	0.13.649	*p* < *0.10*	0.95.986	0.17.644	20	0.13.219	*p* < *0.10*	0.95.973	0.17.472
Maize grain–moisture 17%										
Length	20	0.17.668	*p* < *0.10*	0.94.619	0.31.299	20	0.21.358	*p* < *0.05*	0.91.851	0.09.274
Width	20	0.25.879	*p* < *0.01*	0.85.961	0.00.776	20	0.30.226	*p* < *0.01*	0.85.127	0.00.560
Thickness	20	0.17.205	*p* < *0.15*	0.90.523	0.05.173	20	0.16.023	*p* < *0.20*	0.94.396	0.28.457

Significant values are in [italics].

Taking into account the value (*p*) obtained at the hypothesis level of *p* < *α* = 0.05, the H_0_ should be rejected in favor of the alternative hypothesis H_1_, assuming that the distribution of a given feature is not consistent with the normal distribution. It was similar in the case of the the width of beans in the new method and for three levels of humidity and the thickness of the beans at 17% humidity (Table 1). Hypothesis H_1_, assuming the lack of compliance of the examined characteristic with the normal distribution, was also observed for the width of the corn grain at 13% and 17% humidity. In control tests, the distribution is abnormal for the width of the bean grain at 9% and 13% moisture and thickness at 13% and 17% moisture.

In the case of the remaining results on the geometric dimensions of beans and corn, there was no grounds to reject the null hypothesis H_0_. This is evidenced by the probability values (*p*) of the tests used (Shapiro–Wilk test and the K-S test with Lilliefons correction), which are statistically insignificant (*p* > 0.05).

If the distribution of the physical properties of beans and corn grains was consistent with the normal distribution, parametric tests were used to determine the significance of differences between the average values of these characteristics obtained using different measuring devices. However, if the basic condition for using parametric tests was not met, the nonparametric Kruskal–Wallis test was used.

**Figure 4:**
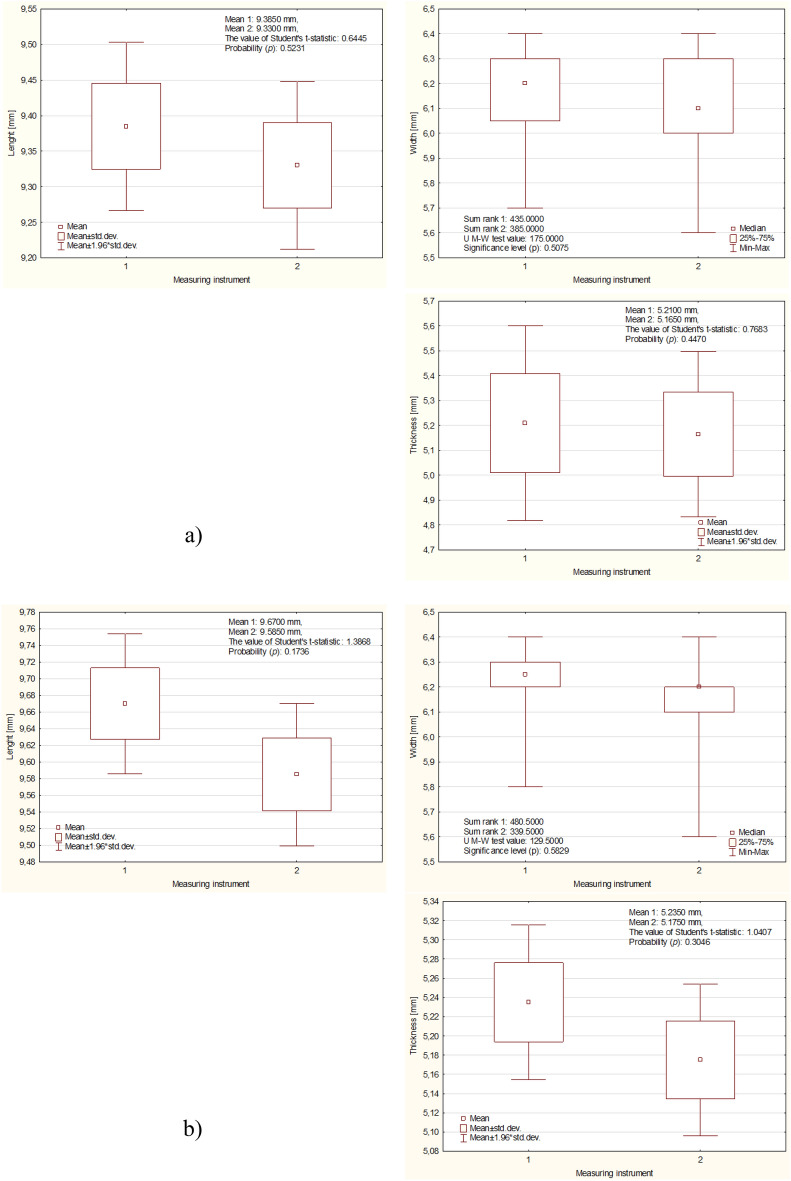

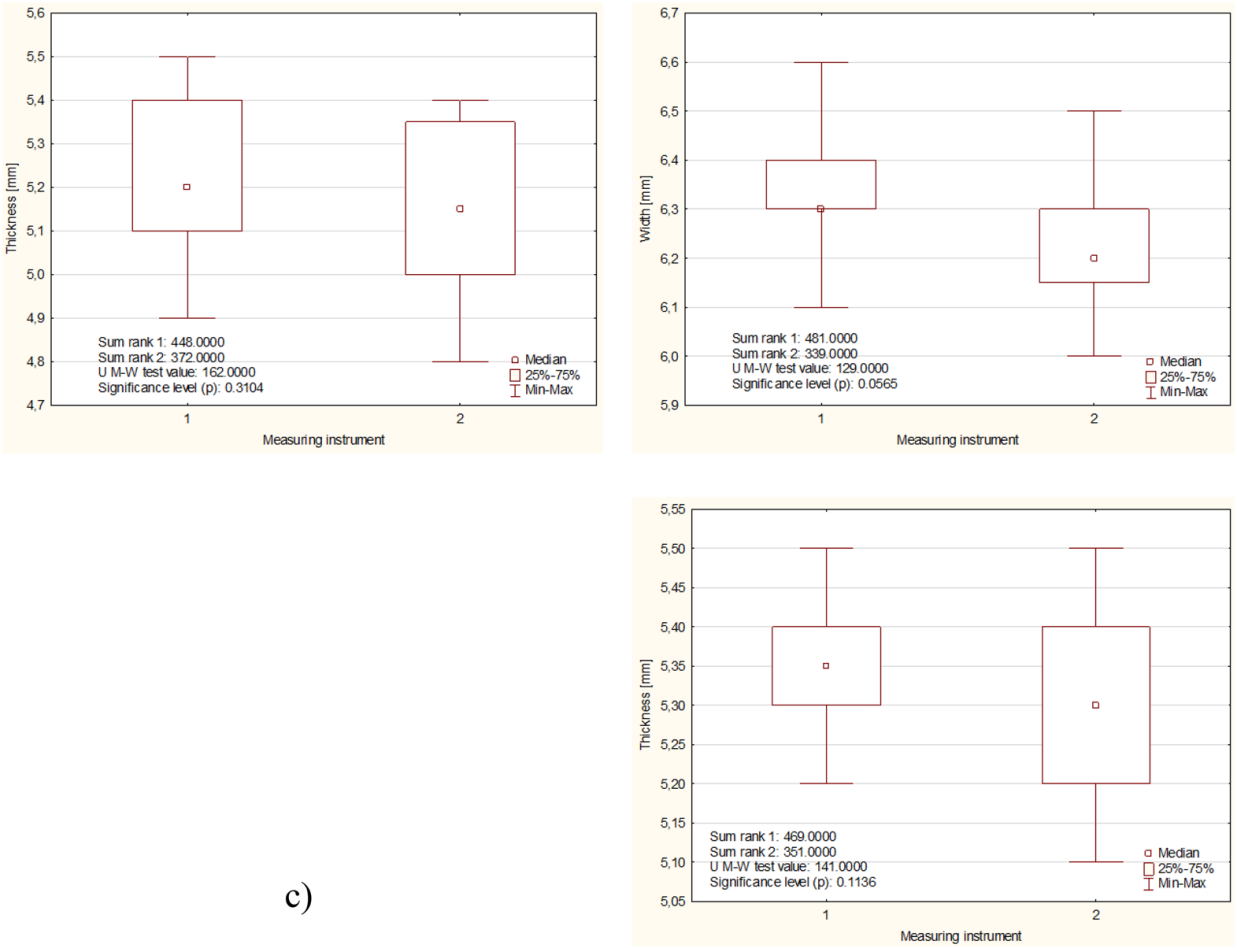
Mean and standard deviation values of fava beans at different moisture levels: (**a**) 9%, (**b**) 13%, (**c**) 17%.

**Figure 5:**
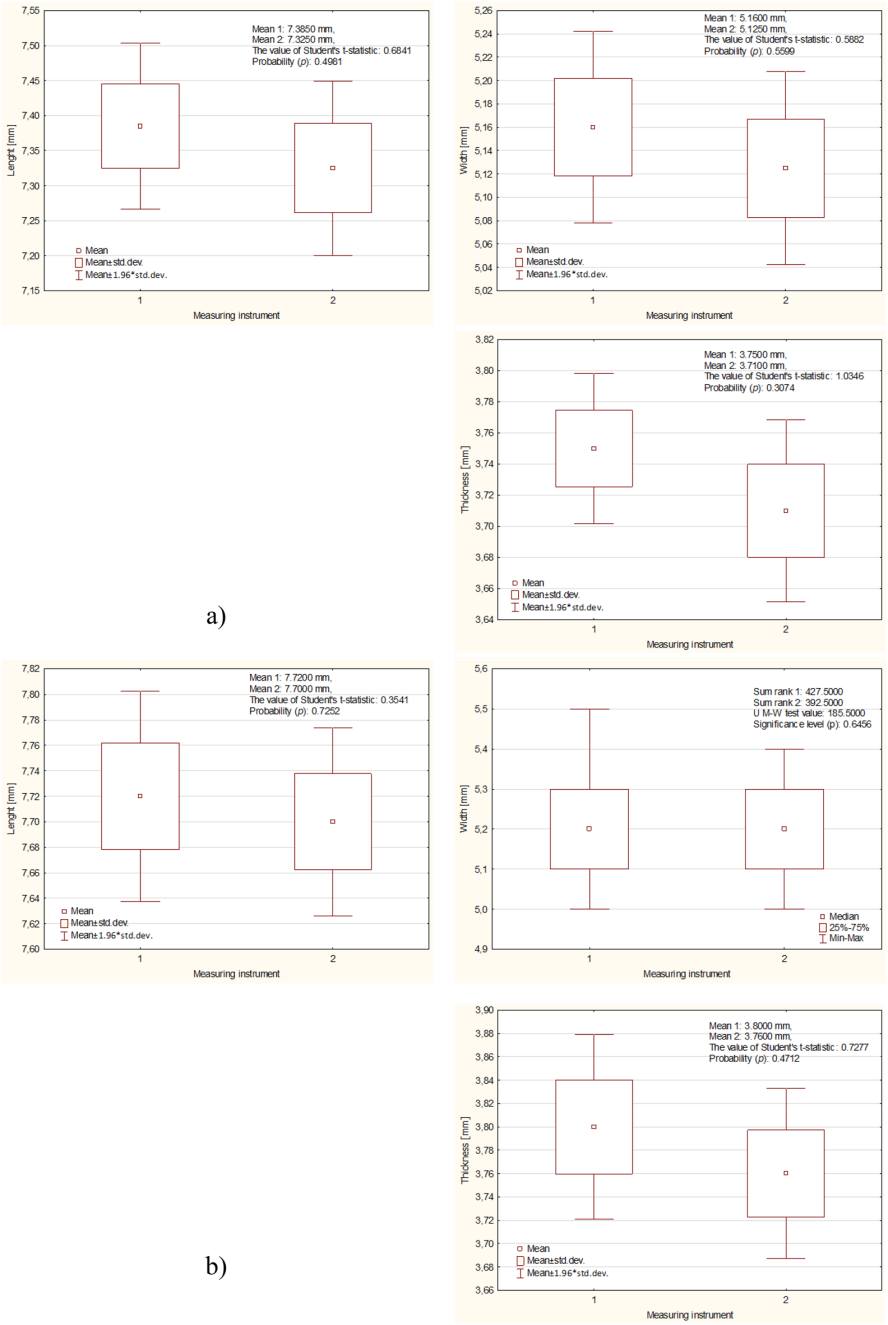

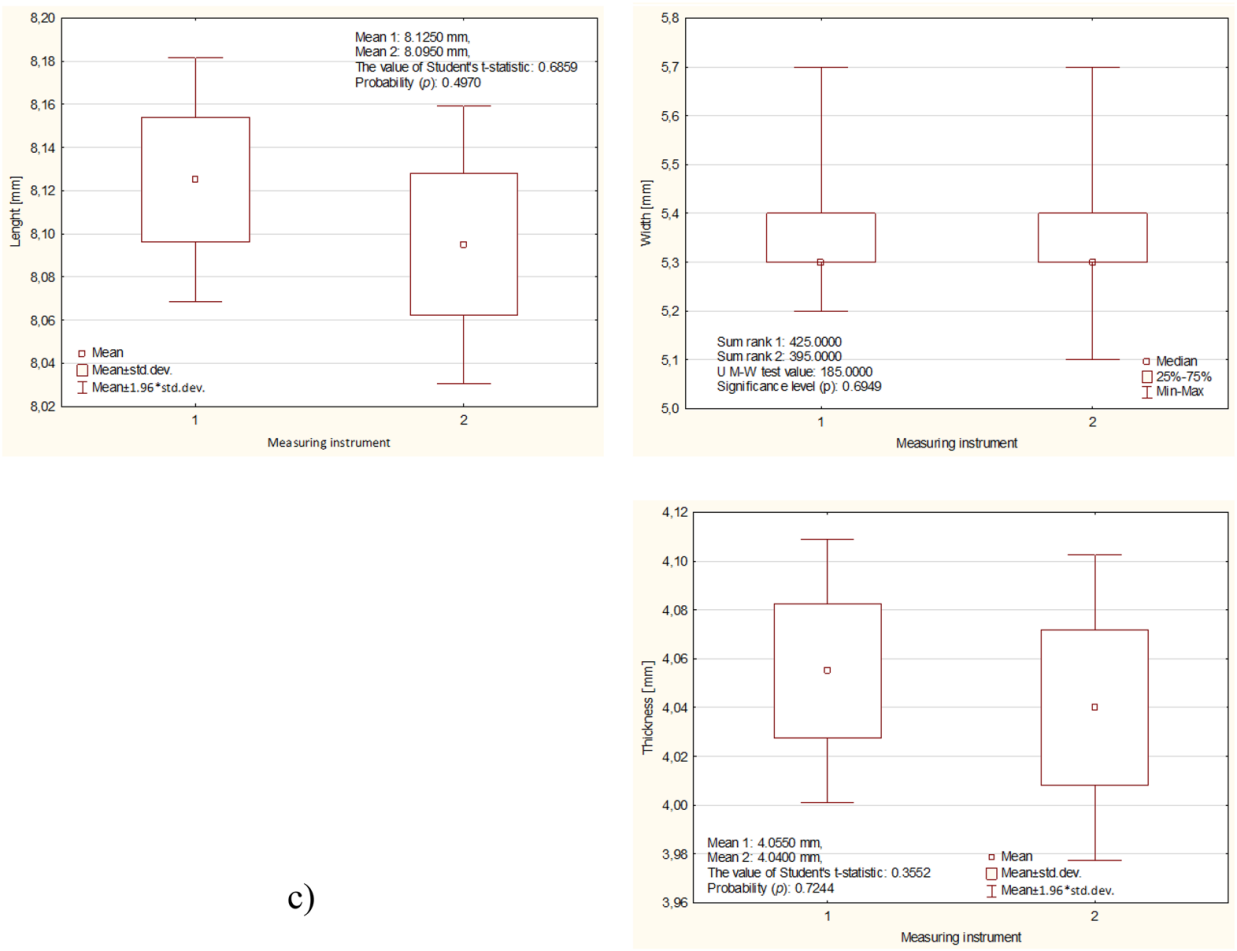
Mean and standard deviation values of fava maize at different moisture levels: (**a**) 9%, (**b**) 13%, (**c**) 17%.

The results of detailed comparative analyzes of the measurement results of the same physical properties of beans and corn, using different measuring instruments, for three levels of grain moisture, are presented in Figs. 4 and 5. As can be seen from the (*p*) value, there was no grounds to reject the hypothesis in any case H_0_ assuming equal values of individual grain dimensions determined using different measuring instruments.

Figure 4 shows the results of the measurements of the physical properties of beans depending on their wet moisture for three different levels (9, 13, 17%), a new measurement method (1), and a caliper (2). The longest kernels (seeds) had dimensions of 9.38, 9.67, 6.25 mm and 9.33, 9.58, 6.20 mm, while the widths of the kernels (seeds) were 6.20, 6.25, 6.30 mm and 6.10, 6.20, 6.20 mm. The high moisture content in the wet state also influenced the grain thickness, as the two previous methods obtained grain thicknesses of 5.21, 5.23, 5.35 mm and 5.16, 5.17, 5.30 mm, respectively.

Figure 5 shows the results of the measurements of the physical properties of corn grains depending on their wet moisture for three different levels (9, 13, 17%), a new measurement method (1), and a caliper (2). The longest kernels (seeds) had dimensions of 7.38, 7.72, 8.12 mm and 7.32, 7.7, 8.09 mm, while the widths of the kernels (seeds) were 5.16, 3.80, 5.30 mm and 5.12, 3.70, 5.30 mm. The high moisture content in the wet state also influenced the grain thickness, as the two previous methods obtained grain thicknesses of 3.75, 3.80, 4.50 mm and 3.71, 3.76, 4.04 mm, respectively.

The new method for analyzing the basic grain dimensions developed in this work turned out to be as accurate as the traditional method based on caliper measurement; therefore, we relied solely on the results of the new method in further analysis.

The results of the analyses (ANOVA and Kruskal–Wallis) showed the appearance of statistically significant differences between the mean values of the individual dimensions of bean and corn grains depending on their wet basis moisture content (*p* < *α* = 0,05)—these are the results marked in red in the table. This was confirmed by ‘post hoc’ research, which allowed us to isolate the so-called homogeneous groups, i.e. those whose mean values do not differ statistically significantly. It should be emphasized that although ‘post hoc’ tests with different "sensitivities" were used to assess the significance of differences between mean values (ANOVA), the final results of the calculations were the same (Table 2 shows sample calculations and the results of Duncan’s test).

**Table 2:** Summary of the calculations results that verify the significance of differences between mean values of bean and maize grins depending on their moisture content on a wet basis.

Bean grain length (Kruskala-Wallisa test) H (2. N = 60) = 35.98052; *p* = 0.0000			
Variable –grain moisture [%]	Probability of multiple comparisons		
	Bean grain–moisture 9% *R* = 14.53	Bean grain–moisture 13% *R* = 29.55	Bean grain–moisture 17% *R* = 47.43
9	–	0.0196	0.0000
13	0.0196	–	0.0036
17	0.0000	0.0036	–

Bean grain width (ANOVA test) Efekt: F(1.056) = 2.2749; *p* = 0.1058			
Variable –grain moisture [%]	Probability Duncan’s tests		
	Bean grain–moisture 9% *X* = 5.7933	Bean grain–moisture 13% *X* = 5.8700	Bean grain–moisture 17% *X* = 5.9800
9	–	0.3835	0.0437
13	0.3835	–	0.2112
17	0.0437	0.2112	–

Bean grain thickness (Kruskala-Wallisa test) H (2. N = 60) = 6.832006; *p* = 0.0328			
Variable –seed moisture [%]	Probability of multiple comparisons		
	Bean grain–moisture 9% *R* = 25.60	Bean grain–moisture 13% *R* = 27.25	Bean grain–moisture 17% *R* = 38.65
9	–	1.0000	0.0544
13	1.0000	–	0.1170
17	0.0544	0.1170	–

Maize grain length (Kruskala-Wallisa test) H (2. N = 60) = 42.15251; *p* = 0.0000			
Variable –grain moisture [%]	Probability of multiple comparisons		
	Maize grain–moisture 9% *R* = 13.75	Maize grain–moisture 13% *R* = 28.48	Maize grain–moisture 17% *R* = 49.28
9	–	0.0230	0.0000
13	0.0230	–	0.0005
17	0.0000	0.0005	–

Maize grain width (Kruskala-Wallisa test) H (2. N = 60) = 16.23271; *p* = 0.0003			
Variable –grain moisture [%]	Probability Duncan’s tests		
	Maize grain–moisture 9% *X* = 22.57500	Maize grain–moisture 13% *X* = 25.97500	Maize grain–moisture 17% *X* = 42.95000
9	–	1.0000	0.0007
13	1.0000	–	0.0063
17	0.0007	0.0063	–

Maize grain thickness (Kruskala-Wallisa test) H (2. N = 60) = 29.40179; *p* = 0.0000			
Variable –grain moisture [%]	Probability of multiple comparisons		
	Maize grain–moisture 9% *R* = 19.550	Maize grain–moisture 13% *R* = 24.68	Maize grain–moisture 17% *R* = 47.28
9	–	1.0000	0.0000
13	1.0000	–	0.0001
17	0.0000	0.0001	–

*X*—mean value (mm); *R*—mean rank.

The dimensional analysis of the grains showed that the higher their moisture content in the wet state, the larger their basic dimensions (length, width, and thickness). From the above results, it was concluded that there is a direct relationship between the moisture content and the main dimensions of the grains. Researchers achieved similar results in their articles^41,42^.

Differences in the nature of changes in the dimensions of bean and corn grains may result from differences between individual grains in terms of water absorption capacity, as well as differences in their chemical composition and morphological structure. The ability of grains to absorb water is important not only during storage, but also during sorting and conditioning before grinding^43–45^. Moisturizing the grains is a technological process that is frequently used. The wheat grain milling technology requires grain with a moisture content of 15.5–16.0%, with storage humidity ranging from 12 to 14%; therefore, in the milling industry, grains are often moistened to the indicated level^46^.

Based on the measurement data, the average values of grain dimensions were compared with the two previous methods to verify the accuracy of the measurement. It was found that the new method of analyzing the main dimensions of grains is as accurate as the traditional method (measurement with a caliper); therefore, the new method can be used to measure the main dimensions of grains and thus predict the moisture content of selected grain species.

### Artificial neural network performance

The ANN of the type of multilayer perceptron (MLP) was used to predict the wet basis moisture content of grains (seeds) based on the main dimensions (length, width, and thickness). The performance of the ANN configuration was evaluated several times using different datasets and configurations. It is best to configure an ANN using two hidden layers, with six cells in the first layer and five cells in the second layer. The correctly classified cases were 19 with a measurement accuracy of 79.2%, a mean absolute error of 0.18, and a mean squared error of 0.34. To determine the network performance measure, the evaluation measures used in the analysis are defined in detail and presented in Table 3.

**Table 3:** Results of network performance determination.

Moisture content on a wet basis %	TP rate	FP rate	Precision	Recall	F-measure	ROC area	PRC area
9	0.83	0.16	0.62	0.83	0.71	0.84	0.78
13	0.82	0.15	0.81	0.81	0.81	0.89	0.90
17	0.71	0.00	1.00	0.71	0.83	0.91	0.89
Weighted avg	0.79	0.11	0.82	0.79	0.79	0.88	0.87

It is clear from Table 3 that the mean positive samples (*TP Rate*) is 0.79. Where the percentage forecast at 9% humidity level gave the highest degree of 0.83. The average positive error (*FP rate*) is 0.11, with the smallest error occurring at 17% humidity. The average percentage of true-form prediction accuracy (*Precision*) was 0.82. Although the *F-Measure* was 0.79, the ROC area was 0.88 and the PRC area was 0.87.

**Figure 6:**
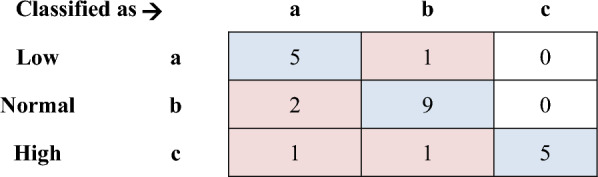
Confusion matrix showing error in prediction.

To better understand the results, a confusion matrix was created that shows the prediction error (Fig. 6). Pink cells show incorrect predictions and blue cells show correct predictions. Where a grain moisture content of 9% is expected, a moisture content of 13% is also expected at one time. A grain moisture content of 13% was predicted to be twice that of 9%. It was also predicted that the grain with a moisture content of 17% would once have a moisture content of 9% and also once have a moisture content of 13%.

**Figure 7:**
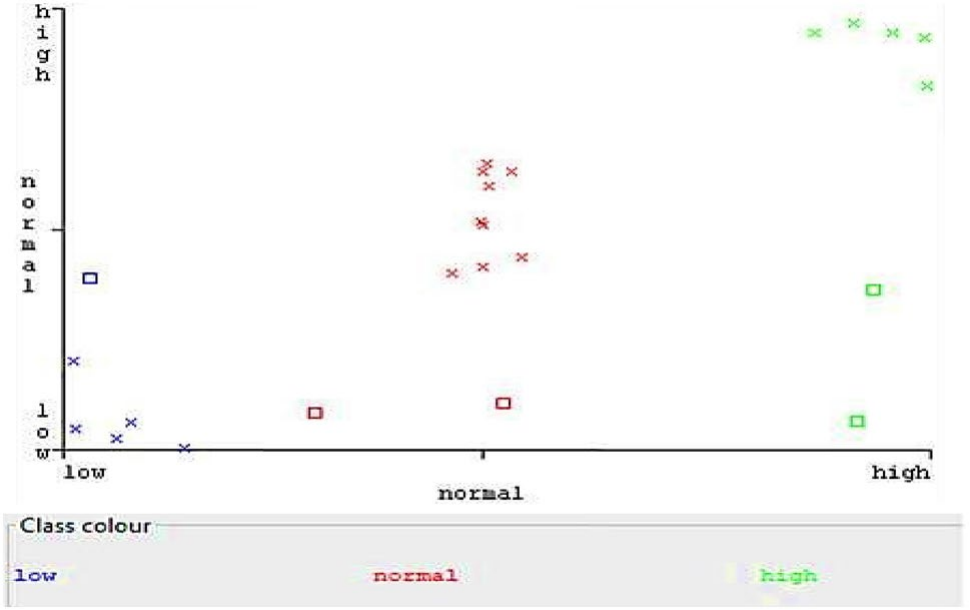
Scatter plot.

The reason for the overlapping results is that the recorded values are very close to each other, which classifies them into one group and, therefore, makes it difficult for the algorithm to predict the indicated humidity value. To illustrate this, the scatter plot shown in Fig. 7. Square-shaped points show results that were incorrectly predicted.

Based on the above results, the neural network to predict the moisture content. Previously, a similar network successfully predicted the sweet taste of oranges^47^. The proposed network can also predict various human diseases^48,49^. Going further, the studied network was used to predict soil penetration resistance^50–52^, as well as soil pH^53^. Neural networks were also used to predict the water quality index^54^.

## Conclusions

In this work, a new system based on image processing technology was developed to measure the main grain dimensions (length, width, and thickness) to determine the effect of wet basis moisture content. The results obtained with the new method were compared with those obtained with the traditional method using a caliper. When examining the normal distribution of the results in the Shapiro–Wilk test for (p = 0.05), the hypothesis of normal distribution was accepted. The innovative approach to grain analysis developed in this work turned out to be as accurate as the traditional method based on measurements made with calipers. Based on the results obtained with the new method, it was found that there is a direct relationship between the wet basis moisture content and the main dimensions of the grains. Additionally, an artificial neural network of the multilayer perceptron (MLP) type was developed to predict the wet basis moisture content of grains based on the main dimensions of the grains. The algorithm consisted of three layers, an input layer representing the main dimensions of the grain, two hidden layers, and an output layer representing the wet basis moisture content of the grains. The first hidden layer consisted of 6 cells, while the second hidden layer consisted of 5 cells. Based on the test results, 19 cases were presented correctly classified with a measurement accuracy of 79.2%, the mean absolute error was 0.18, and the mean square error was 0.34. Furthermore, the proposed artificial neural network gave an efficiency of 82%. We conclude that, on the results of modern technological methods, it is possible to predict wet basis moisture content based on dimensional measurements using an artificial neural network. Due to this, it is possible to predict the dimensions of grains (seeds) based on wet basis moisture content measurement and correctly set the sowing dose, taking into account the volumetric nature of seed dosing using dispensers. Moreover, in further research, we plan, in addition to the use of artificial neural networks, to use other artificial intelligence techniques or hybrid models to predict the dimensions of grains (seeds) based on moisture measurements.

## Data Availability

The datasets used and/or analysed during the current study available from the corresponding author on reasonable request.
